# From Stagnation to Strategy: Challenges in Advancing Long COVID Research

**DOI:** 10.1111/jep.70180

**Published:** 2025-08-13

**Authors:** Appleby Ellen

**Affiliations:** ^1^ Wadham College University of Oxford UK

**Keywords:** complexity of health, healthcare, medical research, public health

## Abstract

**Background:**

Long COVID is a debilitating multisystemic condition and is a major public health burden, yet the pathophysiology remains poorly understood and there are no effective treatments. Despite the urgent need for better management strategies, research into long COVID is losing momentum.

**Objectives:**

To help tackle this loss of momentum, this article analyses the major challenges impeding progress and proposes innovative strategies to navigate them and to reinvigorate this research field.

**Method:**

The analysis of the long COVID research domain drew on a broad range of scientific literature to identify major barriers to research and potential pathways forward.

**Results:**

The research highlighted critical obstacles, including the lack of reliable biomarkers which has necessitated a reliance on symptom reporting that is inherently heterogenous, temporally complex and often confounded by symptoms arising from pre‐existing comorbidities. The absence of pre‐infection baseline data further complicates the distinction between long COVID‐specific pathophysiology and the effects of pre‐existing co‐morbidities. Additionally, the long COVID patient population has heterogenous multiorgan pathology, and this diversity makes it difficult to identify and interpret clinical findings.

**Conclusion:**

Addressing these methodological and conceptual challenges is essential to accelerate the understanding of long COVID pathophysiology and guide the development of effective interventions.

## 1 Introduction

The World Health Organisation defines long COVID as the continuation or development of new symptoms 3 months after the initial severe acute respiratory syndrome coronavirus‐2 (SARS‐CoV‐2) infection, with these symptoms lasting for at least 2 months with no other explanation [1]. Similar to other post‐acute infection syndromes, long COVID symptoms are prolonged and can resolve within 12 months or persist for years after the initial infection [2, 3, 4].

Long COVID is a major public health burden, with the *Office of National Statistics* estimating that 3.3% of people in England and Scotland have long COVID, 74.7% of which have a reduced ability to perform daily activities [5]. Long COVID is not only a health challenge but also a policy challenge, particularly in workforce capacity, equitable access to care and healthcare system financing. Patient advocacy groups, including Long COVID SOS and Long Haul COVID Fighters, have advanced our understanding of its multifaceted nature. The long COVID research domain is vast, with over 5243 articles on PubMed and Web of Science, but the momentum of research is slowing [6]. With the pathophysiology remaining poorly understood and the lack of effective treatments, it is essential that there is reinvigoration in this research space so that we can meet the needs of patients with long COVID and better understand chronic illness associated with infectious disease.

To tackle the loss of momentum in long COVID research, the extensive body of long COVID literature must be critically analysed to identify the factors impeding progress. This articles analysis draws on a broad range of sources and uses selected studies to exemplify the critical challenges hindering research progress. The article finishes with propositions of creative strategies to overcome these challenges with the aim of ultimately improving patient outcomes.

## 2 Why Is Measuring Symptoms Flawed?

The lack of validated clinical, imaging and metabolomic biomarkers in long COVID research has led to a reliance on symptom‐based surveillance. The use of symptom‐based approaches hinders advancement because long COVID presents as a broad array of symptoms with variable nature which makes accurate characterisation of each patient error prone. An international cohort study involving 3762 long COVID participants reported an average of 55.9 symptoms across 9.1 organ systems. [7] This data is at risk of sampling bias owing to participant self‐selection, however, an independent study also reported a vast quantity of symptoms with 42% of 201 community‐based long COVID patients having 10 or more symptoms [8]. Secondly, long COVID symptoms are nonspecific, such as fatigue and brain fog, and overlap with common co‐morbidities making it difficult to identify long COVID specific symptoms from exacerbations of pre‐existing pathology. Thirdly, there is no standardised symptom measurement tool which limits cross study comparability because different tools have different sensitivities.

Another challenge in measuring symptoms arises from their complex temporal patterns: they can persist from, or develop after, acute SARS‐CoV‐2 infection and wax and wane. Therefore, infrequent cross‐sectional measurement risks missing important symptoms. Studies also correlate pathophysiology with limited symptom documentation. For instance, a 2022 double‐blind trial involving 73 long COVID patients reported that hyperbaric oxygen therapy caused limbic microstructural changes linked to improved psychiatric symptoms. [9] In this study symptoms were assessed twice – at baseline and after the last oxygen therapy – using a survey which evaluates the average severity of symptoms over the prior 7 days. Such study designs do not fully capture temporal fluctuations or symptoms that present outside the brief assessment window, which can make it challenging to robustly correlate symptom changes with underlying pathophysiology.

A further challenge arises from the high rates of cognitive and mental health impairments in long COVID patients, which could compromise the reliability of patient reporting, potentially leading to inaccurate or inconclusive results. A 2024 systematic review reported that 20% of 41,000 long COVID patients had a mental health condition or brain fog. [10].

## 3 How Does Heterogenous Pathology Obstruct Advancement?

**Figure 1:**
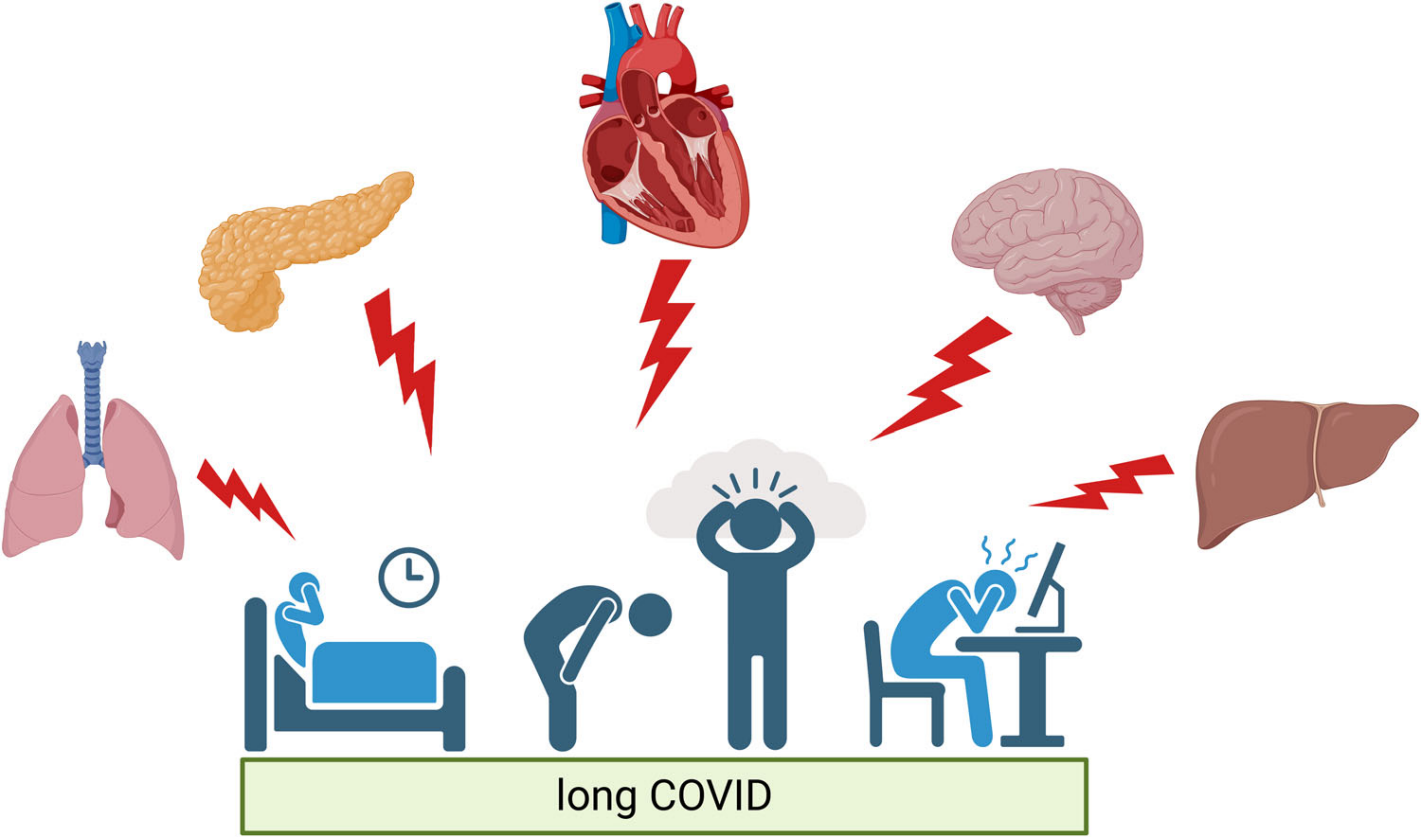
Multiorgan pathology is a feature of long COVID. Created in BioRender [12].

Consistent with the heterogeneity of symptoms, long COVID is associated with organ pathology that is different between patients, as shown in Figure 1. The extent of the pathophysiological heterogeneity was demonstrated by a 2021 prospective study that used 201 long COVID patients to report MRI‐detected mild organ impairment in the pancreas (40%), heart (26%), lungs (11%) and liver (28%) and that 29% had multiorgan impairment. [8] Heterogenous organ pathology is complex to control for, particularly because the extent of pathophysiology in long COVID is not well understood. Hence, poorly controlled heterogenous cross‐system dysfunction between patients can confound the interpretation of measured outcomes. In particular, it is likely that heterogenous long COVID related immune dysregulation, including systemic inflammation, can confound the measured outcomes of studies [11]. Concerningly, poorly controlled systemic pathophysiology could lead to the misattribution of outcomes to unrelated factors by correlation.

The heterogeneity in pathophysiology extends beyond differences in organs to differences within dysfunctional organs. A 2022 cross‐sectional study demonstrated this by identifying two clusters using a deep learning model on lung CT scans from 140 long COVID patients: one cluster had increased air‐trapping caused by small airways disease (*p *= 0.008) while the other had decreased lung volume (*p* < 0.001) and increased ground glass opacity (*p* < 0.001). [13] Heterogenous pathophysiology complicates the identification of mechanisms important to specific subgroups of patients. Furthermore, there is no consensus on long COVID subtypes which makes it challenging to extrapolate findings to the appropriate patient populations.

## 4 Why Aren't Homogeneous Groups of Patients Studied?

Stratifying long COVID patients into more homogenous subgroups would facilitate the understanding of specific long COVID presentations which would lead to a better understanding of the heterogenous pathophysiology and more personalised patient care. The identification and validation of subtypes is challenging due to the need for large cohorts to capture patient heterogeneity, the expense of deep phenotyping, and multiple confounding factors affecting measured outcomes.

Importantly, long COVID subtypes identified by symptom‐based studies do not often overlap, which makes it difficult to interpret the results. Table 1 demonstrates subtypes identified by independent studies using electronic health records. The divergence of findings could indicate that symptom‐segregated subtypes are not distinct, their symptom data is low quality or that their sample was not representative of the long COVID population. Interestingly, Dagliati et al. reported that the neurological deficit subtype had symptoms appearing 1 month earlier than other subtypes. [14] This suggests that symptom documentation timing may bias subtype identification and contribute to inconsistencies between studies. This further illustrates the complexity of studying long COVID using symptoms.

**Table 1:** Independent studies have identified different long COVID subtypes using symptom data.

	Dagliati et al. (2023) [14]	Zhang et al. (2022) [15]	Reese et al. (2022) [16]
Number of participants	12,424	20,881	2256
Subtype 1 sequelae	Chest pain	Cardiac and renal	Cardiovascular
Subtype 2 sequelae	Dyspnoea	Respiratory, sleep and anxiety	Pulmonary
Subtype 3 sequelae	Neurological deficit	Musculoskeletal and nervous system	Neuropsychiatric
Subtype 4 sequelae	Cognitive changes	Digestive and respiratory	Multisystem sequelae and multiple laboratory abnormalities
Subtype 5 sequelae	Chronic malaise and fatigue		Pain and fatigue
Subtype 6 sequelae	Joint pain		Multisystem sequelae and pain
Subtype 7 sequelae	Dyspepsia		

Studies that use metabolomic data to investigate subtypes also suffer from inconsistent results. This could stem from the utilisation of small sample sizes, which are commonplace owing to the cost of acquiring metabolomic data. Differing hospitalisation rates and pre‐existing conditions between study populations also contribute to inconsistent results. For example, a 2024 multicentre study analysed 368 plasma proteins in 426 long COVID patients and reported that complement and myeloid inflammation were common to all subtypes. [17] Conversely, another study identified inflammatory and noninflammatory subtypes by investigating 2925 protein features in 97 long COVID patients. [18] Both sample sizes were small and cohort differences may confound the measured outcomes, with the former study including only hospitalised patients and they found pre‐existing conditions associated with all identified subgroups.

## 5 Why Is This Patient Population Particularly Difficult to Study?

The risk and symptom profile of long COVID are influenced by many factors, including the SARS‐CoV‐2 variant, [19, 20, 21] vaccination status, [19, 22, 23] age, [24] sex, [19, 25, 26] comorbidities [19, 25, 27] and hospitalisation during acute infection. [28, 29] Importantly, these factors can affect pathophysiology and thus confound results and contribute to inconsistencies between studies. For example, a 2024 cross‐sectional study conducted immune profiling on 165 participants with and without long COVID and reported that male and female participants had different symptoms, organ system involvement and immune features, [30] which suggests sex can bias results.

It is methodologically complex to disentangle the effects of multiple confounding factors on pathophysiology. The following study illustrates how confounding factors complicate result interpretation. Grist et al. (2022) studied breathless long COVID patients and reported gas transfer differences between patients hospitalised with acute infection (*n *= 12) compared to those not hospitalised (*n *= 11). [31] These differences may be caused by critical care myopathy, due to immobilisation and mechanical ventilation, or by drug side effects. However, the hospitalised patient group were significantly older, which confounds the attribution of intergroup differences to hospitalisation. Age‐related reductions in lung compliance, vascularity and injury tolerance may have influenced outcomes. This example illustrates how the interpretation of studies is complicated by heterogenous patient characteristics.

The heterogenous characteristics are difficult to control for in studies and can influence how representative the sample population is of the long COVID population. Given that the acute SARS‐CoV‐2 infection can be asymptomatic and that asymptomatic acute infection can lead to long COVID, there is a considerably likelihood that people have long COVID yet have not received a diagnosis [32, 33]. This patient population are likely to be underrepresented in trials and hence the pathophysiology of long COVID in this patient group less well understood.

## 6 Why Does the Scarcity of Pre‐Infection Data Matter?

The scarcity of pre‐infection baseline data limits the ability to control for pre‐existing pathology, which can lead to the misinterpretation of findings as manifestations of long COVID. This particularly hinders research because approximately two thirds of long COVID patients have chronic comorbidities. [34]

Longitudinal data sources that include pre‐infection baselines are crucial for understanding changes that associate with long COVID. Wearable devices provide data collected non‐invasively before, during and after acute SARS‐CoV‐2 infection, which enables the study of effects associated with long COVID. For example, a 2021 cohort study used data from Fitbit watches to report that 13.7% of 234 people had elevated heart rates 133 days postinfection, [35] which suggests lasting cardiovascular changes associate with infection. Importantly, the accuracy of watch data can vary with activity [36] and external factors—such as the stress of a pandemic—could confound the data. The UK Biobank COVID‐19 Repeat Imaging Study provides pre‐ and postinfection organ scans and is a valuable resource for understanding long COVID. For example, a 2022 study used this data to report greater brain size reductions in infected participants (*n *= 401) compared to uninfected controls (*n *= 384), [37] which suggests dynamic brain changes can occur following SARS‐CoV‐2 infection. The Repeat Imaging Study's target sample size of 2000 is too small to capture the extent of patient heterogeneity. [38] The UK Biobank's data is also limited by their focus on participants over 40 and a self‐selection bias that may underrepresent severe long COVID. Pre‐infection data from electronic health records is another resource, but it is biased towards individuals with pre‐existing conditions, frequent healthcare use and the ability to access healthcare.

## 7 Navigating the Challenges

### 7.1 More Advanced Study Designs Will Facilitate the Identification of Biomarkers

Biomarkers could offer a quantitative, objective method for disease monitoring and hence they could improve patient care and study comparability, compared to the use of symptoms. Furthermore, biomarkers could provide a complementary indicator of pathophysiology, when combined with symptoms, to understand the effectiveness of treatment interventions. The investigation of accessible biomarkers should be prioritised to facilitate their use in research and clinical practise. Systematic reviews are essential for validating biomarkers because large sample sizes are required to capture the broad patient heterogeneity.

**Figure 2:**
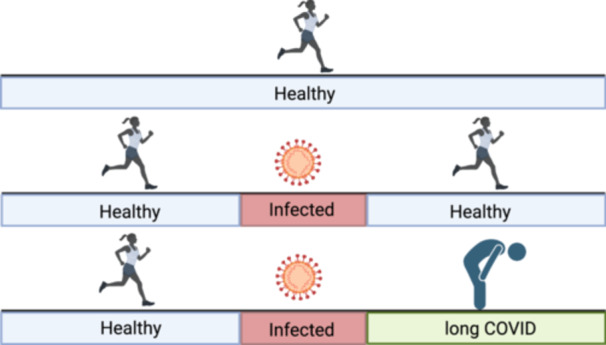
Groups that should be used in pathophysiology research. The healthy group controls for the levels of the measured outcome in health. The recovered SARS‐CoV‐2 infected group controls for the effects of SARS‐CoV‐2 infection outcome. Created in BioRender [40].

Despite extensive biomarker research, progress is hindered by study designs that don't control for the effects of pre‐existing conditions or acute infection recovery. Study designs that include infection recovery can disentangle specific acute infection effects from long COVID effects. A recent systematic review reported significant associations between serum biomarkers and specific long COVID symptoms. [39] However, only 4 of the 28 studies included both healthy uninfected controls and recovered infected participants. The study design, illustrated in Figure 2, would aid the disentanglement of effects of long COVID from pre‐existing conditions or acute infection recovery and if it were to be used more it would increase study comparability. In practise, this study design is challenging to recruit for because long COVID has an age dependency and there is a decreasing prevalence of disease‐free individuals with age. After making discoveries using this study design, subsequent research should investigate the outcomes specificity and value in patients with more advanced comorbidities.

### 7.2 Statistical and Machine Learning Approaches Can Disentangle the Complexity of Long COVID

Multiple factors confound the interpretation of long COVID results so statistical modelling should be used to understand their effects on pathophysiology. Klein *et al* (2023) showed the value of this approach by using linear models to associate long COVID with the levels of nonconventional monocytes after adjusting for age, sex and body mass index. [41] Statistical modelling relies on well‐characterised participants which poses challenges for its use in large cohort studies due to financial constraints.

Machine learning analysis is already being used on large data sets, including electronic health record data, to investigate subtypes of long COVID patients [15]. A strength of this study method is that it can analyse complex data sets, involving a vast number of participants and multiple variables, which are particularly vital in this field owing to the heterogenous nature of the long COVID patient population. Further use of this approach could help to better characterise long COVID subtypes which would enable the study of more homogenous patient groups and facilitate more personalised approaches to patient care.

### 7.3 Symptomatic Data Should be Obtained Using Standardised Questionnaires and Smartphone Apps

Self‐reported symptoms should inform research because they reflect patient experiences and offer insights into pathophysiology. To improve study comparability a standardised symptom questionnaire should be developed that is informed by narratology studies. The utility of narratology studies is elaborated upon in Panel 1.

Panel 1 **Utilising narratology studies.**

1 Narratology studies interview patients and document their experience of disease. In contrast to surveys, they collect information in a more authentic way that highlights nuanced disease manifestations. A 2021 study interviewed 114 long COVID patients for 45 to 90 min and captured profound experiences of disease. [42] Patients described exhaustion so profound that they could barely move. A participant that works as a doctor said “2 days ago I couldn't remember the word brain. I described it as a thing that was like a blancmange in your head, the weirdest thing”. Such accounts highlight symptoms that most severely affect patients' lives and should be prioritised in quantitative survey assessment.

Infrequent surveys miss symptoms that wax and wane. To address this, longitudinal cohort studies should use smartphone apps to continuously collect data on symptom type and severity. Frequent symptom measurement would increase accuracy because symptom recall is over a shorter duration. A 2023 longitudinal study successfully used a smartphone app to gather detailed symptom data over 14 days, with five daily entries, and identified within‐subject symptom correlations. [43] The study focused on only seven symptoms which suited the intensive data collection period. Future designs should balance the frequency of data collection with the duration to complete the survey to maintain patient compliance. Importantly, this method may underrepresent older age groups. Overall, longitudinal cohort studies that continuously monitor symptoms with the ease of data collection via smartphone apps could improve our understanding of symptom fluctuation and disease progression.

### 7.4 Data Should be Collated in a Centralised Repository

Large sample sizes are essential to capture diverse clinical presentations, pathophysiology and the heterogenous characteristics of long COVID. In response, the international scientific community should create a centralised online repository for long COVID data. This resource would enable specialists to analyse the same data using different techniques, encourage collaboration and a holistic understanding of the multisystemic pathology.

An online repository would also optimise the use of scarce pre‐infection data. Aggregated pre‐infection datasets could be analysed with machine learning to identify trends that correlate with long COVID and dissociate them from pre‐existing co‐morbidity flare ups. This data could be powerful in advancing our understanding of causative disease mechanisms. Machine learning on the centralised data repository could also advance our understanding of long COVID subtypes.

Governments should fund detailed phenotyping of patients including longitudinal symptom measurement, MRI organ scans, spirometry and omics analyses (including proteomics and metabolomics). Omics analysis offers key molecular insights into long COVID pathophysiology and combining this with research techniques that examine pathophysiology from a wider lens, such as MRI imaging, would promote a more cohesive understanding of the complex multisystem pathophysiology. Precision phenotyping on a large scale would also promote collaboration between scientists that are experts in diverse research approaches which would further facilitate a more holistic understanding of long COVID.

## 8 Conclusion

Long COVID is a major public health burden that affects millions, and the pathophysiology remains poorly understood. The reliance on symptom documentation, which is narrow in scope and lacks temporal detail, highlights the need for smartphone apps to track symptom fluctuations and for validated biomarkers. Mathematical modelling offers a solution for evaluating the effects of confounding factors on pathophysiology. A centralised data repository would facilitate the use of machine learning and holistic research approaches. Overall, advancement depends on researchers recognising these challenges and adopting innovative and collaborative strategies to navigate them.

## Conflicts of Interest

The author declares no conflicts of interest.

## Patient and Public Involvement

Neither patients, nor the public, were involved in this research. Upon shadowing a doctor in a long COVID clinic I learnt how the needs of long COVID patients were not being met which inspired me to research into why.

## Data Availability

The author has nothing to report.
